# Data-driven challenges and opportunities in crystallography

**DOI:** 10.1042/ETLS20180177

**Published:** 2019-07-26

**Authors:** Calina Glynn, Jose A. Rodriguez

**Affiliations:** Department of Chemistry and Biochemistry, UCLA-DOE Institute for Genomics and Proteomics, University of California, Los Angeles (UCLA), Los Angeles, CA 90095, U.S.A

**Keywords:** cryoEM, crystallography, diffraction, electron microscopy, serial crystallography

## Abstract

Structural biology is in the midst of a revolution fueled by faster and more powerful instruments capable of delivering orders of magnitude more data than their predecessors. This increased pace in data gathering introduces new experimental and computational challenges, frustrating real-time processing and interpretation of data and requiring long-term solutions for data archival and retrieval. This combination of challenges and opportunities is driving the exploration of new areas of structural biology, including studies of macromolecular dynamics and the investigation of molecular ensembles in search of a better understanding of conformational landscapes. The next generation of instruments promises to yield even greater data rates, requiring a concerted effort by institutions, centers and individuals to extract meaning from every bit and make data accessible to the community at large, facilitating data mining efforts by individuals or groups as analysis tools improve.

## Introduction

The crystallographic analysis of macromolecules is increasingly dependent on data-driven approaches [1]. Growing data output is a central feature of the new tools that facilitate crystallographic analysis. These include ultra-bright ultra-fast X-ray sources, upgraded synchrotron facilities and accessible instruments for routine electron diffraction. Single experiments can now yield gigabytes or terabytes of data from one or more samples allowing unprecedented exploration of ultra-fast processes or from increasingly small crystallites [2–4]. Here, we discuss two areas of rapid growth in structural biology that are increasingly reliant on large-scale data gathering: serial X-ray crystallography [1,5,6] and electron crystallography of micro and nanocrystals [7–10]. While other structural biology approaches are without a doubt also reliant on large datasets [11], data demands in crystallography are rapidly growing. We focus on these two closely related crystallographic approaches and their evolution from conventional data gathering to large-scale data collection and analysis.

While the two crystallographic approaches we highlight are somewhat similar, some key differences are important to note. Most of the X-ray crystallography measurements we describe are performed in a small number of large, government-funded facilities with staff and computational infrastructure matched to the scale of the experiment. In contrast, electron diffraction efforts are generally driven by individual groups and are implemented across a large number of small-scale facilities. Nonetheless, data collected by each approach is largely sparse and must be reduced to a small set of measurements for structure determination. The focus of this review is, therefore, the challenge of data reduction and the extraction of information from large sets of individual diffraction patterns belonging to one or more crystals.

**Figure 1.**
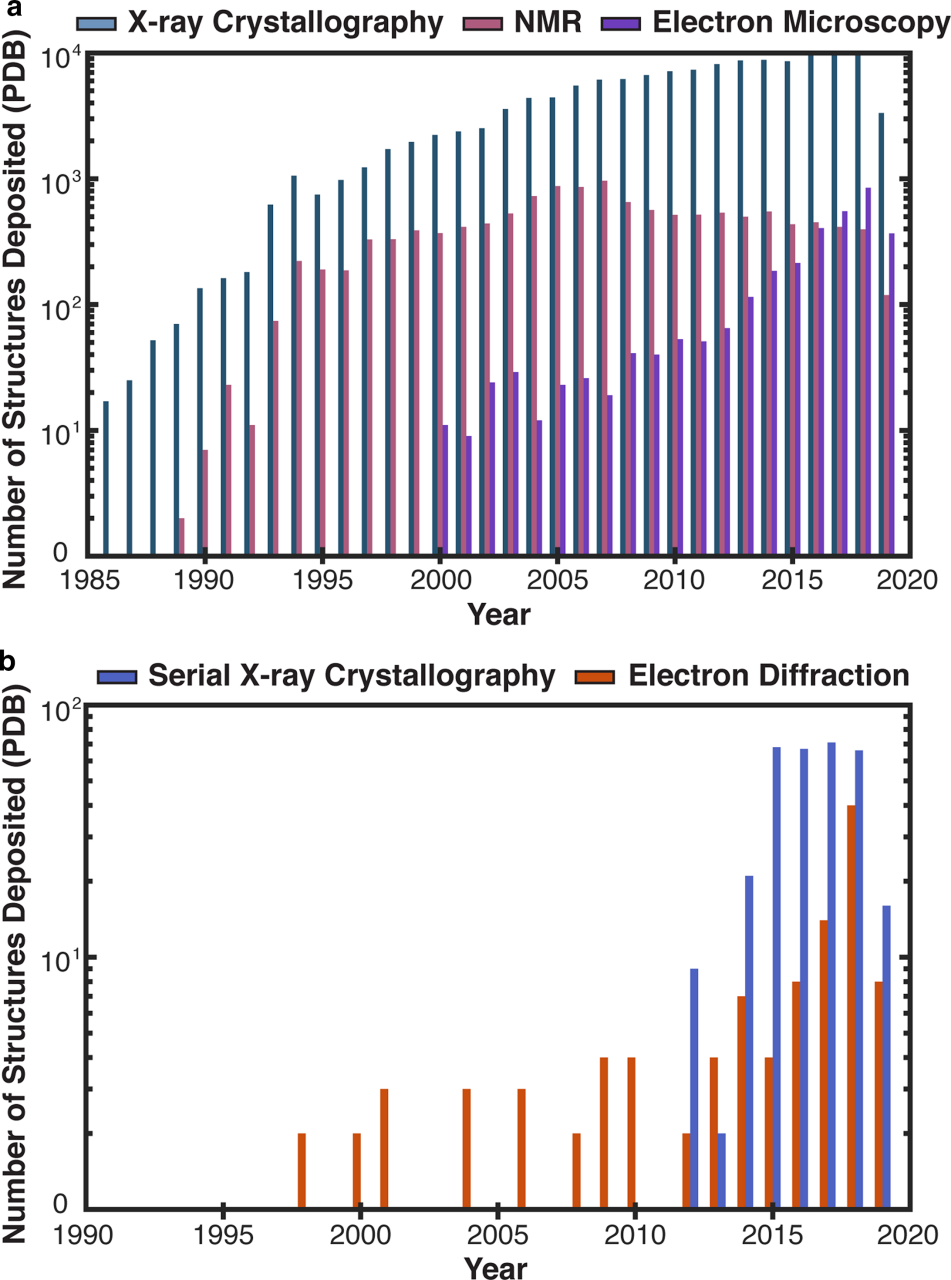
Number of structures deposited in the PDB per year [20]. (**a**) Structures deposited per year from 1985 to 2019 associated with all X-ray crystallographic methods, compared with nuclear magnetic resonance (NMR) and electron cryo-microscopy (cryoEM). Each counted structure is associated with X-ray crystallography (blue), NMR (pink) or electron microscopy (magenta). (**b**) A subset of structures deposited per year from 1990 to 2019 associated with serial X-ray crystallography or electron crystallography, including MicroED. Each counted structure is associated with data collection by serial X-ray crystallography methods, including those acquired at X-ray free electron laser facilities (purple), compared with those acquired by electron crystallography methods (orange).

Conventional X-ray crystallography efforts set the stage for the analysis of this type of data by establishing methods for routine data collection and processing from macro-scale crystals. The success of these approaches has yielded a consistently high number of structures deposited in the protein data bank (PDB) per year, a number that remains unmatched by any other structural biology method (Figure 1). Likewise, efforts in electron cryo-microscopy (cryoEM) [11–19] have established analogous pipelines for structure determination [11,19] that directly benefit electron crystallographic approaches [8], giving rise to a growing number of deposited structures per year (Figure 1) as catalogued by the protein data bank (PDB) [20].

The rise in structures determined by these new methods is accompanied by increasing challenges associated with storage, analysis and accessibility of data [20–23]. The Electron Microscopy Public Image Archive has been established to facilitate the public archival of electron microscopy and diffraction data [22]. Likewise, data repositories like the coherent X-ray imaging data bank are providing resources for depositing the terabytes of data collected at X-ray free electron laser facilities [23]. As the use of fast detectors grows routine in crystallography, the number of sites worldwide collecting large datasets will place additional demands on these and analogous resources.

## Advances in X-ray diffraction

**Figure 2.**
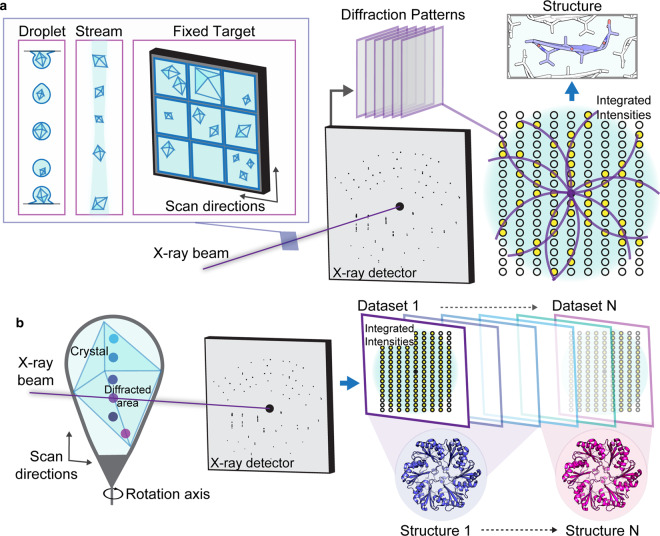
Examples of data gathering by serial X-ray crystallography. (**a**) Ultra-fast pulses of high energy X-rays are directed at a sample interaction region to which samples can be delivered by varying means. Three methods of sample delivery are pictured: droplet-based delivery, stream or liquid-jet delivery, and fixed target delivery, typically by a windowed device. Data from a train of patterns recorded from different crystals are combined to yield one or more datasets that are used to determine a molecular structure. (**b**) Approaches that pursue multiple structures from a single crystal can train an X-ray beam on particular locations on a crystal, collecting multiple datasets that can then be combined or individually yield a structure. In this way, several structures can be obtained from a single crystal or a set of similar crystals. This example illustrates determination of the monoclinic structure of EutL, PDB ID 4TLH.

The advent of high brightness sources for crystallographic study of macromolecules took a quantum leap with the development and introduction of ultra-bright, pulsed X-ray lasers [6,24,25]. While initially developed with the goal of determining structures of single molecules, acquiring a signal before inducing a coulombic explosion of each molecule [26,27], X-ray lasers have opened new doors for the crystallographic study of proteins and nucleic acids [1,6,28]. The first structure published by this approach was that of photosystem I, for which more than 3 million diffraction patterns were collected [2]. Among the first demonstrations of the feasibility of this approach was an effort to demonstrate the routine ability to determine structures of proteins from crystal slurries (Figure 2). This was demonstrated with the structure of lysozyme determined from a slurry of microcrystals [29], although many other sample delivery strategies have since been demonstrated [30] (Figure 2). This new kind of experiment, termed serial femtosecond crystallography (SFX) [2,6], allows the detection of high-resolution diffraction from individual microcrystals micrometers on edge. Lysozyme crystals were delivered to the 9.4 keV X-ray beam via a focused jet of liquid with a 10-micrometer focus, and out of 1.5 million individual diffraction frames collected, about 12 000 were indexed and integrated to yield a high-resolution structure [29]. Diffraction frames measured the interaction of crystals with individual 40 or 5 femtosecond pulses at a rate of 120 Hz, yielding a data stream of gigabytes per minute [29]. This approach has now been applied to a whole variety of samples, including entirely unknown structures, some of whose crystals are grown in cells [31–38]. A key feature of this data-driven crystallographic approach is the ability to probe dynamics, capturing the atomic scale motions within proteins [28,32,39,40].

The latest generation of ultra-fast X-ray sources offers even greater throughput [41]. The megahertz X-ray free electron laser facility, part of the European XFEL project, delivers 10 pulse trains of megahertz X-ray sequences delivering up to 27 000 pulses per second [41]. However, efficiently utilizing a torrent of pulses presents a challenge for real-time processing and data storage. Diffraction produced by 50-pulse trains impinging on crystals in a liquid jet every 100 milliseconds was captured by a megapixel Adaptive Gain Integrating Pixel Detector [41,42]. A jet with flow rates of ∼50 m/s allows the fresh sample to be probed by the series of pulses in each train and the recording of individual diffraction patterns within a train, with pulses in a train separated by ∼900 ns [41].

Serial femtosecond crystallography inspired the adaptation of methods and technologies for serial data collection efforts at synchrotron beamlines [43–45]. There, millisecond timescale shutterless detection of the interaction between focused X-ray beams and a high viscosity crystal matrix or crystals held in fixed target mounts has facilitated routine room-temperature determination of structures from thousands of diffraction patterns within minutes to hours [43–46]. The large, fast detectors used in some of these experiments accelerate data collection [47]; 18-megapixel images collected at high frame rates (50 Hz) allow the collection of a single dataset in under 90 min [44]. Collection of room-temperature data at this rate in an accessible synchrotron beamline has enabled high-resolution structure determination [45,48], including determination of structures *de novo* and with bound ligands [44]. Acquisition frame rates of 50 Hz appear to improve the signal to noise, particularly at high resolution by allowing the collection of multiple diffraction patterns per crystal and the elimination of background from empty frames [44]. This was particularly advantageous for *de novo* structure determination through the improved measurement of the anomalous signal [44]. Importantly, information about ligand binding could be acquired from as few as 900 images (9 Gb) collected over a period of 5 min [44]. This opens the door to large-scale screening efforts for ligand binding at dedicated serial crystallography beamlines. This may be even further enabled by the development of compact X-ray sources that deliver high-repetition bright X-ray beams in a facility with the footprint of a conventional laboratory [49].

A different type of serial crystallography experiment uses bright X-ray sources to expand the amount of data collected from single crystals and extract more information out of each (Figure 2) [48,50,51]. These strategies train an X-ray beam on particular regions of a crystal or collections of crystals, collecting a partial or full dataset at each location (Figure 2). The data collected at each location can then be indexed and integrated, and the data between sampled locations correlated to yield multiple datasets per crystal [51]. This approach is especially advantageous when radiation damage is a challenge, and helps explore the possible conformational heterogeneity present within macro-scale crystals that may represent structural variations in their constituent proteins [52]. In the simplest case, regions within a crystal can be non-isomorphous and distinguished by varying unit cell parameters and described by a lattice distortion [51]. Combining data within isomorphous sets then allows for a single structure to be determined per set (Figure 2), a comparison of the structures reveals conformational differences that explain the lattice distortion [51]. With greater data sampling, the potential to mine differences in crystal structures in search for conformational heterogeneity becomes increasingly possible. These approaches, generally referred to as multiple structures from one crystal (MSOX), are not limited to spatial heterogeneity in crystals, but can also add a temporal component to assess changes induced by radiation [50,53] or changes due to environmental differences or molecular actions [54].

## Advances in electron diffraction

**Figure 3.**
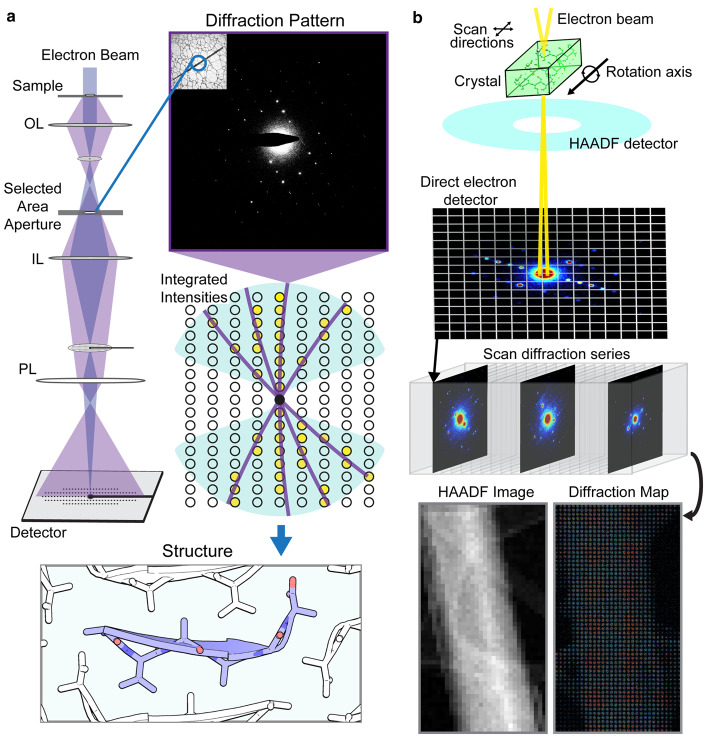
Examples of data gathering by selected area or scanning electron diffraction. (**a**) In a conventional electron diffraction experiment, a crystal and its surroundings are illuminated with a low-dose electron beam. A selected area aperture samples diffraction from a portion or all of the illuminated crystal; diffraction patterns are sampled continuously as the crystal is unidirectionally rotated within the electron beam. Integrated intensities from these patterns populate a reciprocal lattice that is phased to determine a molecular structure. OL, objective lens; IL, intermediate lenses; PL, projection lenses. (**b**) In scanning nanobeam electron diffraction, a focused electron beam is scanned across a sample while diffraction patterns are recorded. Electrons scattered to high angle at each scan point are integrated by a dark field detector (HAADF) to yield a visual representation of the sample, while electrons scattered to a lower angle travel through a hole in that detector and are recorded by a fast readout, pixelated electron counting detector to yield a diffraction map of the scanned region. Both panels show data collected from nanocrystals of a hexapeptide.

Improvements to detector technology have played a major role in the interrogation of macromolecular structures from thin three-dimensional crystals by dramatically increasing the rate of accurate data gathering [9,10,55]. The field of electron crystallography of three-dimensional microcrystallites (MicroED) is a marriage of cryoEM sample manipulation and data collection with X-ray crystallography analysis for routine structure determination of macromolecular structures [8]. The MicroED technique, initially demonstrated on the well-known protein standard lysozyme [9], has now been successful in determining a variety of structures from small molecules to macromolecular assemblies (Figure 1) [56–58]. To obtain electron diffraction from 3D protein crystals, approaches like MicroED illuminate a sub-micron thick crystal with an extremely low-dose electron beam (Figure 3). The signal from the illuminated crystal can be isolated from that of its surroundings by means of a selected area aperture (Figure 3). Diffraction is then sampled from the selected area and recorded either as a movie from a continuously rotating crystal (Figure 3) [59] or as a series of precession photographs at discrete tilt angles [60]. Data is reduced by conventional crystallography approaches to yield integrated intensities that can cover part or all of the reciprocal lattice, depending on crystal symmetry and the fraction of Bragg reflections obstructed by the supporting grid and holder (Figure 3). Structures are determined from this data either by direct methods [61–63], when the resolution of the data is atomic (∼1.2 Å) or by molecular replacement when a suitable probe exists [8]. Over one hundred macromolecular structures have now been deposited in the PDB, determined by electron diffraction since 1990 (Figure 1). Nearly half of these are determined from 3D protein crystals, and add to a growing number of small molecule structures [64–67].

While the number of structures deposited into the PDB by MicroED is miniscule in comparison with X-ray crystallography efforts (Figure 1), growth in the number of sites collecting MicroED data and the implementation of automation will pose a challenge for efforts promoting archival and access to raw data. This challenge is most prominent for diffraction data collected on fast electron detectors [3,47], especially where multiple datasets must be combined to yield information about a single structure. The simplest of these experiments is one where data from several tilt series is merged to create a single high completeness dataset [8,68], but even this effort can be a challenge since the specific combination of datasets that yields the best merge is not known *a priori*. In cases where polymorphism is high, and diffraction quality is highly variable between crystals, obtaining a quality merge may require a collection of data from dozens or more individual crystals. Widespread adoption of this approach necessitates methods for automation in data collection and processing [69,70]. Partial automation is already facilitating more complex experiments, including scanning nanobeam diffraction where thousands of patterns are collected at a given orientation from a single crystallite (Figure 3) [3,71], and serial crystallography experiments that combine diffraction from hundreds or thousands of crystallites using SerialED software [69].

Scanning diffraction experiments are particularly data rich [3]. In a scanning electron diffraction experiment, a focused electron beam is positioned at specific locations across a sample in a defined pattern (Figure 3), analogous to the experiments performed when scanning an X-ray beam across a much larger crystal (Figure 2) [51]. While the beam is positioned at each location for only a small fraction of a second, a diffraction pattern is collected on a fast detector [72]. Data volume in scanning diffraction scales varies with the number of individual scan steps. Depending on the size of a scan area and the density of locations sampled, this procedure can generate tens of thousands of diffraction patterns per scan, multiplied by the number of crystallite tilt angles sampled (Figure 3). Data in each scan is plentiful, but information is sparse; each diffraction pattern in a scan has few Bragg reflections, and each reflection may only be partially sampled [3]. This makes averaging over similar patterns advantageous. Ultimately, all patterns containing a usable signal could be combined into a single set of integrated intensities representing a subset or the whole of reciprocal space. By spatially linking reciprocal space information to specific locations on a crystal, scanning diffraction offers a rich source of information unavailable in a conventional crystallography experiment [3,51]. While a simple analysis of total diffracted intensity can reveal regions of strong or weak diffraction within a crystal, more subtle changes in the diffracted signal can be extracted by algorithms that compare deviations across patterns. Comparison of patterns across a scan can ultimately reveal changes in lattice orientation [3], and potentially changes in unit cell parameters or perhaps changes in the structure of molecules in a crystal.

## The role of detectors in big data gathering

The use of advanced detectors for photon and electron counting is expediting data collection in both X-ray and electron crystallography, facilitating the interrogation of crystals with short exposure times using high-flux sources. An outstanding challenge is posed by the rapid readout Adaptive Gain Integrating Pixel Detector [42], a system capable of delivering bursts of 350 images with a 6.5-mHz frame rate [41,73]. This and other fast detectors are now key to enabling new efforts in serial crystallography [43], where their high dynamic range and sensitivity allow rapid readout from single X-ray pulses or short electron beam exposures [43,47,74].

Electron microscopy as a whole has largely benefitted from the development of advanced detectors, first by the introduction of slow readout charged couple device (CCD) sensors [75], then by faster active pixel sensors including the complementary metal-oxide semiconductors (CMOS) including direct electron detectors [76,77]. Each improvement in detector technology has grown data output, facilitating not only improved speed and accuracy of data collection but also more efficient data archival and processing [77]. While the readout rates of several electron detectors are now in the kilohertz range [65,74,76], most experiments are not performed in this regime. Fast readout is instead used to preserve resolution in electron microscopy images by limiting the influence of motion induced artifacts [78]. In continuous rotation electron diffraction, readout rates are adjusted to limit overexposure due to brightly diffracted beams, limit readout noise and ensure adequate sampling of the reciprocal lattice (Figure 3) [8,68,74].

Performing electron diffraction of single crystals at kilohertz frame rates could allow the collection of a 140-degree wedge of reciprocal space while sampling a quarter of a degree per frame in approximately half a second. However, the use of direct electron detectors for measuring diffraction from macromolecular crystals not only facilitates rapid, low-noise, low-dose data collection but can also increase the data rate per experiment by over an order of magnitude. This startling pace of acquisition would have a dramatic effect on our ability to rapidly screen samples, probe heterogeneity in crystallites and ultimately sample more experimental parameters. However, the collection of 14-megapixel, 8-bit images at 400 frames per second yields a torrential 5.6 gigabytes of raw data per second. In line conversion of this data from its archival format to one suitable for crystallographic analysis is the first of many bottlenecks. Since crystallographic data is highly sparse, the greatest improvement to processing of kilohertz diffraction will come from algorithms for rapid and automated data reduction [68,69].

## Toward real-time processing

Algorithms for accurate data reduction from sparse diffraction data are key enabling technologies for emerging crystallographic experiments. Rapid hit-finding and indexing algorithms are routinely used in X-ray free electron laser facilities during serial crystallography experiments [79–85], where the rate of image collection far outpaces the amount of data that can be parsed manually in real time. Algorithms used to parse 120 Hz data generated by the Cornell-SLAC Pixel Array Detector can operate concurrently with data collection, giving the crystallographer a notion of how many frames have usable diffraction and how many reflections are present in a frame [81,83–86]. Many of these same packages can create and refine experimental parameter models, index reflections and integrate reflection intensities. Programs such as KAMO [87] and Blend [88] facilitate the identification of consistent unit cells and evaluate the capacity of data subsets to be merged. Their algorithms rely on the relationships between cell parameters or intensity correlations to select suitable merge sets. Whilst a similar feat of automated data processing has only recently been demonstrated in electron diffraction [69]. Although no fundamental hurdles limit the full applicability of these methods to continuous rotation, serial or nanobeam electron diffraction, sparse single electron diffraction patterns may be especially difficult to index without sufficient prior constraints on cell dimension and experimental geometry. The need for a centralized access to a comprehensive set of tools and protocols for analysis of large crystallographic datasets will continue to be underscored by the continued and rapid development of sources, instruments, methods, programs and algorithms [89].

## Summary

- New crystallographic approaches are facing new challenges and opportunities delivered by data-rich experiments facilitated by the development of new ultra-fast detectors.
- Electron crystallography experiments are following a similar trend, where faster detectors are allowing the rapid collection of information-rich data from single crystals or collections of crystals.
- New algorithms for automated data collection and analysis are leveraging the rise in data production to yield faster structure solutions, and new insights into the macromolecular structure at the time and storage space limits.

## Abbreviations

CCD: charged couple device
CMOS: complementary metal-oxide semiconductors
MSOX: multiple structures from one crystal
MicroED: microcrystallites
SFX: serial femtosecond crystallography
